# Retraction: Evaluating the biological characteristics of targeted ZIF-8-encapsulated individual and combined drug systems for enhanced *in vivo* toxicity mitigation using folic acid ligands

**DOI:** 10.1039/d6ra90103d

**Published:** 2026-11-09

**Authors:** Noor S. Sadeq, Mas Jaffri Masarudin, Mohd Basyaruddin Abdul Rahman, Suet Lin Chia, Syahida Ahmad, Haslina Ahmad

**Affiliations:** a Department of Chemistry, Faculty of Science, Universiti Putra Malaysia, UPM 43400 Serdang Selangor Malaysia haslina_ahmad@upm.edu.my noor.s.sadeq@uotechnology.edu.iq; b Medical & Industrial Materials Branch, Applied Science Department, University of Technology Baghdad Iraq; c Department of Cell and Molecular Biology, Faculty of Biotechnology and Biomolecular Sciences, Universiti Putra Malaysia Serdang 43400 Selangor Malaysia; d Nanomaterials Synthesis and Characterisation Laboratory, Institute of Nanoscience and Nanotechnology, Universiti Putra Malaysia Selangor Malaysia; e Integrated Chemical BioPhysics Research, Faculty of Science, Universiti Putra Malaysia (UPM) Serdang 43400 Selangor Malaysia; f UPM-MAKNA Cancer Laboratory, Institute of Bioscience, Universiti Putra Malaysia Serdang 43400 Selangor Malaysia; g Department of Microbiology, Faculty of Biotechnology and Biomolecular Science, Universiti Putra Malaysia, UPM 43400 Serdang Selangor Malaysia; h Department of Biochemistry, Faculty of Biotechnology and Biomolecular Science, Universiti Putra Malaysia, UPM 43400 Serdang Selangor Malaysia

## Abstract

Retraction of ‘Evaluating the biological characteristics of targeted ZIF-8-encapsulated individual and combined drug systems for enhanced *in vivo* toxicity mitigation using folic acid ligands’ by Noor S. Sadeq *et al.*, *RSC Adv.*, 2026, **16**, 1912–1931, https://www.doi.org/10.1036/D5RA07756G.

We, the authors, hereby wholly retract this article due to errors in the interpretation of the FTIR data in Fig. 1 and the reliability of the survival data in Fig. 8.

Having consulted with independent experts, the *Royal Society of Chemistry* has determined that any changes to the paper would be major, and, therefore, that the best course of action is retraction.

We, the authors, are happy with the decision to retract this article.

Signed: Noor S. Sadeq, Mas Jaffri Masarudin, Mohd Masyaruddin Abdul Rahman, Suet Lin Chia, Syahida Ahmad and Haslina Ahmad

Date: 5^th^ November 2026

Retraction endorsed by Laura Fisher, Executive Editor, *RSC Advances*

